# Successful treatment of 1 patient with chlorine-induced ARDS using awake self-prone positioning and nasal high-flow oxygen: A case report

**DOI:** 10.1097/MD.0000000000036995

**Published:** 2024-01-19

**Authors:** Fugui Wang, Fangfang Liu, Houqing Lu

**Affiliations:** a Department of Intensive Care Medicine, Tongling People’s Hospital, Anhui Province, P. R. China.

**Keywords:** acute respiratory distress syndrome, awake self-prone positioning, case report, chlorine gas, high-flow nasal oxygen, treatment

## Abstract

**Rationale:**

Accidents involving chlorinated compounds in the context of cleaning are not uncommon. However, improving the treatment success rate for acute respiratory distress syndrome (ARDS) patients caused by chlorine gas presents significant challenges.

**Patient concerns:**

A 28-year-old female was admitted to the intensive care unit after accidental inhalation of chlorine gas resulting in ARDS.

**Diagnoses:**

The patient was diagnosed with ARDS attributed to chlorine gas exposure.

**Interventions:**

The intervention involved utilizing a combination of awake self-prone positioning (ASPP) and high-flow nasal oxygen therapy for treatment.

**Outcomes:**

After continuous ASPP and high-flow nasal oxygen therapy, the patient quickly recovered and was transferred out of the intensive care unit on the 6th day without any adverse events.

**Lessons:**

ASPP combined with high-flow nasal oxygen therapy can improve patients’ hypoxemia, prevent the need for intubation, avoid rapid deterioration of the condition, reduce treatment complexity, and lower mortality rate.

## 1. Introduction

Cleaning agents and bleaching agents often contain chlorinated compounds, leading to the risk of chlorine gas exposure in cleaning accidents. Inhalation of chlorine gas primarily affects the respiratory tract, potentially causing upper respiratory tract irritation, acute lung injury, and even acute respiratory distress syndrome (ARDS).^[1]^ Typical symptoms include difficulty breathing, coughing, and severely ill patients may require high-flow oxygen therapy or even mechanical ventilation.^[2]^ The current focus of clinical treatment is to improve the success rate of treatment for ARDS caused by chlorine gas exposure and avoid the need for mechanical ventilation.

Existing evidence has shown that awake self-prone positioning (ASPP) combined with high-flow nasal oxygen (HFNO) therapy can reduce the need for intubation and has good patient tolerance with a low incidence of adverse reactions in patients with mild to moderate ARDS caused by pneumonia.^[3]^ However, there are currently no reports on the use of this treatment method in ARDS patients caused by chlorine gas exposure. In August 2023, we successfully applied ASPP in combination with HFNO therapy to treat a patient with chlorine gas-induced ARDS. The details are reported as follows.

## 2. Case presentation

The patient is a 28-year-old female who first used disinfectant tablets containing dichloroisocyanuric acid and trichloroisocyanuric acid produced by a certain factory at around 7:30 am on August 9, 2023. While dissolving the disinfectant tablets in water by stirring, the patient experienced symptoms of chest tightness, wheezing, eye pain, and mild dizziness, but did not lose consciousness. The patient sought medical attention at the emergency department, and hospitalization was recommended, but the patient’s family refused and opted for treatment with methylprednisolone for inflammation and cefdinir for infection. Subsequently, the patient was transferred to an external hospital and received 2 sessions of hyperbaric oxygen therapy, but the symptoms did not significantly improve. In the afternoon of the same day, around 5:00 pm, the patient visited our hospital again and was admitted. Chest CT scan showed extensive infiltration in both lungs.

**Figure 1.**
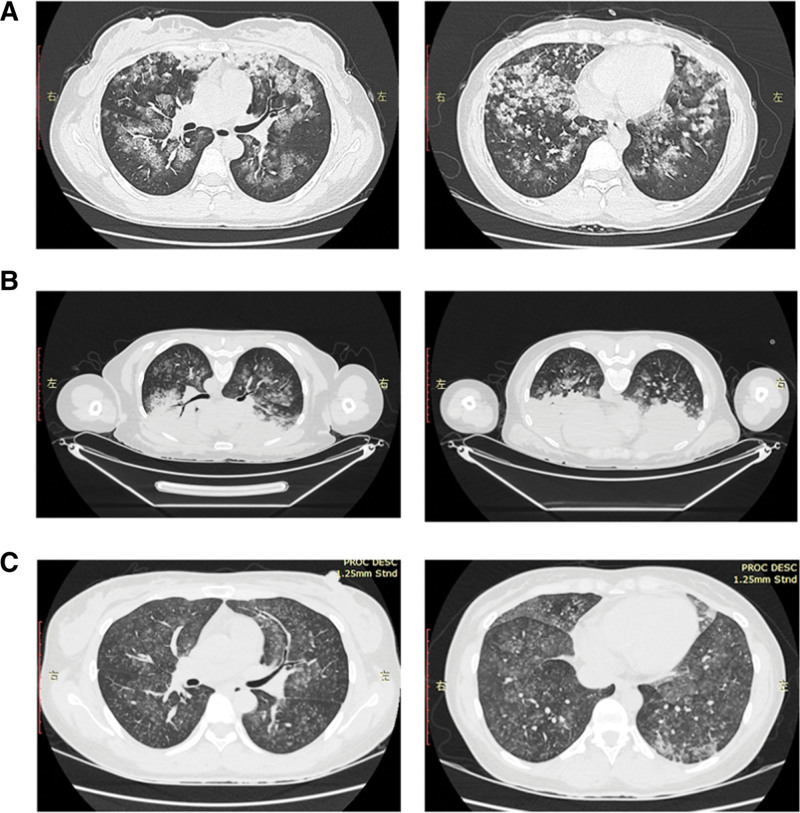
Chest CT scan findings of the patient. (A) On admission, the Chest CT scan indicated extensive infiltration in both lungs. (B) On the third day after admission, the chest CT scan showed diffuse inffltrative lesions, focal consolidation, and atelectasis in both lungs. (C) On the sixth day after admission, the chest CT scan showed significant absorption of diffuse inffltrates in both lungs. CT = computed tomography.

At 8:00 pm that evening, the patient was transferred to the intensive care unit (ICU). The patient had a history of good health, with no history of smoking or alcohol consumption. Physical examination revealed a body temperature of 36.6°C, pulse rate of 118 beats per minute, respiratory rate of 20 breaths per minute, and blood pressure of 103/89 mm Hg. The patient was alert but appeared distressed, with significant moist rales heard upon lung auscultation, a heart rate of 118 beats per minute with regular rhythm, and no apparent abnormalities found during abdominal examination. Further examinations performed after admission include a complete blood count, which showed a white blood cell count of 16.21 × 10^9^/L, lymphocyte count of 0.54 × 10^9^/L, neutrophil percentage of 95.2%, and platelet count of 225 × 10^9^/L. Coagulation profile revealed a fibrinogen level of 2.98 g/L and a D-dimer level of 4.91 mg/L. Stool examination did not reveal any abnormalities. Blood gas analysis showed a pH of 7.37, a partial pressure of carbon dioxide of 38.5 mm Hg, and a partial pressure of oxygen of 103.0 mm Hg. Chest CT scan indicated extensive infiltration in both lungs, as shown in Figure 1A.

**Figure 2.**
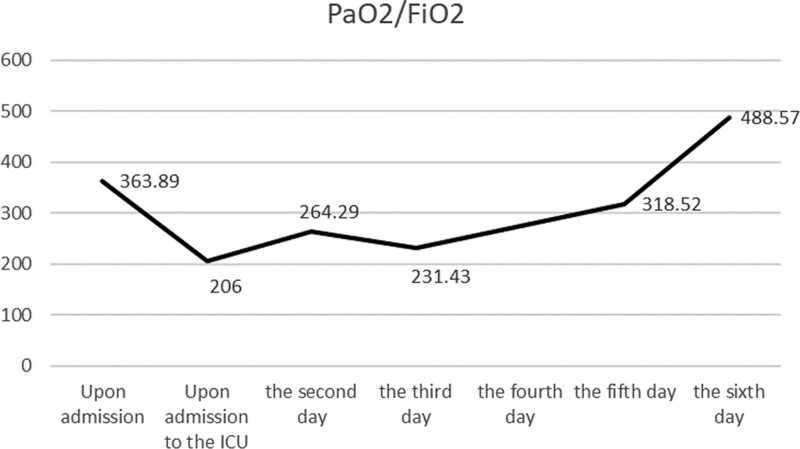
Trend of examination results during patient’s treatment. FiO_2_ = fraction of inspired oxygen, ICU = intensive care unit, PaO_2_ = partial pressure of arterial oxygen.

The patient presented with symptoms of chest tightness and wheezing after inhaling irritating gas, accompanied by eye pain and dizziness. Chest CT scan upon admission revealed extensive infiltration in both lungs, indicating an acute onset with an oxygenation index of <300 mm Hg. As the patient’s pulmonary edema couldn’t be explained by heart failure or fluid overload, a diagnosis of chlorine gas poisoning, chemical pneumonia, and ARDS was made. After transfer to the ICU, the patient received continuous cardiac monitoring, ASPP, and HFNO (oxygen flow rate of 40 L/min, oxygen concentration of 40%). Additionally, the patient was treated with cefpodoxime-sulbactam for infection, dexamethasone and blood purification for anti-inflammation, nebulization, organ protection, and maintenance of a stable internal environment. On the 3rd day, a follow-up chest CT scan showed diffuse infiltrative lesions, focal consolidation, and atelectasis in both lungs (Fig. 1B). The patient was already placed in the awake prone position and started intermittent lateral positioning. On the 4th day, the patient was encouraged to intermittently lie supine, actively cough and expectorate to promote sputum clearance, and received ambroxol hydrochloride orally. On the 5th day, the patient’s breathing stabilized under high-flow oxygen therapy, and a repeat blood gas analysis showed near-normal results. The parameters for high-flow oxygen therapy were gradually reduced. On the 6th day, a follow-up CT scan showed significant absorption of diffuse infiltrates in both lungs (Fig. 1C). The repeat blood gas analysis results showed a pH of 7.42, pressure of carbon dioxide of 40.2 mm Hg, pressure of oxygen of 171.0 mm Hg, and the patient’s oxygenation index gradually returned to normal (Fig. 2). After discontinuation of HFNO, the patient was transferred out of the ICU and continued treatment in a general ward.

## 3. Discussion

Chlorine gas is a common halogen irritant extensively used in household and industrial chemicals. Accidental exposure to chlorine gas can occur in home and occupational settings, such as industrial accidents, improper use of household cleaning agents, swimming pool chlorination accidents, as well as chemical warfare and terrorist attack events.^[4]^ Chlorine gas has moderate water solubility and reacts with respiratory tract mucus upon inhalation, forming hydrochloric acid and hypochlorous acid. Hypochlorite salts, which are oxidation products of chlorine gas, play a significant role in the action of chlorine gas.^[5]^ Injury to the lungs caused by chlorine gas can lead to respiratory distress, hypoxemia, airway obstruction, pneumonia, pulmonary edema, and ARDS, as well as adverse effects on cardiovascular, neurological, ophthalmic, and dermatological systems. The effects of chlorine gas are dose-dependent, with concentrations ranging from 1 to 30 ppm resulting in mild to moderate mucous membrane irritation and concentrations exceeding 30 ppm potentially exhibiting mild clinical respiratory features. Concentrations of 40 to 60 ppm can cause pneumonia and pulmonary edema, while concentrations above 400 ppm may even lead to death within minutes to 30 minutes.^[6]^

ARDS is a severe disease characterized by increased pulmonary capillary permeability, resulting in acute hypoxemia and bilateral pulmonary edema. According to the morphological classification, ARDS can be divided into the early exudative phase and the late fibroproliferative phase, with the former presenting diffuse alveolar, interstitial, and alveolar wall injuries, and the latter exhibiting interstitial fibrosis.^[7]^ From a physiological perspective, prone positioning helps to reexpand dorsal alveoli and reverse atelectasis. Additionally, ASPP achieves more uniform ventilation, reducing lung inflation heterogeneity caused by changes in thoracic pressure and distribution, thereby promoting ventilation/perfusion matching and reducing shunt.^[8]^ ASPP is an adjunctive ventilation strategy originally proposed by Douglas et al^[9]^, aiming to improve the oxygenation index of acutely respiratory failure patients who remain awake and conscious. The results of a prospective observational cohort study conducted by Ding et al^[10]^ showed that the early use of ASPP combined with HFNO or noninvasive ventilation, especially for patients with mild to moderate ARDS and baseline oxygen saturation >95%, can delay or avoid the need for endotracheal intubation. HFNO therapy is a treatment method that provides patients with high concentrations of oxygen to improve oxygenation. It effectively corrects hypoxemia by delivering continuous and stable oxygen flow while alleviating the burden of inadequate ventilation. In cases of severe lung injury caused by chlorine gas, HFNO can provide sufficient oxygen to meet the needs of damaged lung tissue and improve oxygenation, relieving respiratory distress. Moreover, by offering positive pressure support to maintain alveolar recruitment, prevent alveolar collapse, and reduce the occurrence of pulmonary edema, HFNO has a positive impact on preventing intubation.^[11]^ Therefore, the comprehensive treatment approach combining ASPP and HFNO provides a hopeful and effective treatment option for patients with ARDS.

In this case, the main active ingredients in the disinfectant tablets were dichloroisocyanuric acid and trichloroisocyanuric acid. When dichloroisocyanuric acid and trichloroisocyanuric acid dissolve in water, they undergo a series of chemical reactions leading to toxicity. Dichloroisocyanuric and trichloroisocyanuric react with water molecules, resulting in the release of chlorine gas and derivatives of isocyanic acid.^[12]^ These gases and derivatives are released into the surrounding environment. When inhaled, they can cause severe irritation to the respiratory system. Chlorine gas reacts with water in bronchial cells, releasing hydrochloric acid and hypochlorous acid, leading to bronchospasm and lung damage.^[13]^ The manifestations of poisoning may vary depending on the concentration of inhaled gas and the duration of exposure.^[14]^ Common symptoms include respiratory distress, cough, chest pain, sore throat, and dyspnea. Severe poisoning can result in pulmonary edema, respiratory failure, and even death. Therefore, it is crucial to follow the instructions for use, handle and store such chemicals carefully to prevent ingestion or inhalation.

Based on the successful treatment experience in this case, the key lies in providing timely respiratory support to patients experiencing respiratory distress and declining oxygenation. ASPP combined HFNO is critical for treating such patients. ASPP helps improve ventilation/perfusion matching and reduces shunt. HFNO does not interfere with patient communication, provides warm and humidified gas, reduces dead space ventilation, promotes sputum dilution and clearance, and protects respiratory cilia, thereby enhancing patient comfort. The combined use of these 2 treatment methods improves patient tolerance, alleviates respiratory distress symptoms, and reduces patient suffering. Therefore, the comprehensive treatment strategy of employing ASPP combined with HFNO is an effective approach in similar circumstances.

## 4. Conclusion

Inhalation of chlorine gas can easily trigger the occurrence of ARDS. Therefore, while administering anti-infection and anti-inflammatory treatments early in the course, close monitoring of patient’s pulse oxygen fluctuation and timely repeat blood gas analysis are required to assess the patient’s oxygen balance status. When hypoxemia symptoms occur, the prompt application of ASPP combined with HFNO is crucial for improving the patient’s oxygenation and preventing the need for intubation, avoiding rapid deterioration of the condition, reducing the difficulty of treatment, and lowering mortality rates. It is worth noting that although the combination of HFNO and ASPP has shown good results for patients with ARDS caused by chlorine gas in this case, it may not be applicable in all situations. Physicians need to consider the patient’s condition and physiological characteristics comprehensively before deciding to implement this treatment method and formulate the optimal treatment plan by combining it with other therapeutic approaches. By making full use of HFNO and ASPP, we can better save the lives of patients and provide more effective strategies for the management of ARDS.

## Acknowledgments

Thanks for this patient who consented to publish her case.

## Author contributions

**Conceptualization:** Houqing Lu.

**Funding acquisition:** Houqing Lu.

**Investigation:** Fangfang Liu.

**Writing – original draft:** Fugui Wang, Fangfang Liu.

**Writing – review & editing:** Fugui Wang, Houqing Lu.
